# Prenatal care and uptake of HIV testing among pregnant women in Gambia: a cross-sectional study

**DOI:** 10.1186/s12889-020-08618-4

**Published:** 2020-04-15

**Authors:** Sanni Yaya, Olanrewaju Oladimeji, Kelechi Elizabeth Oladimeji, Ghose Bishwajit

**Affiliations:** 1 School of International Development and Global Studies, Faculty of Social Sciences, University of Ottawa, 120 University Private, Ottawa, ON K1N 6N5 Canada; 2 The George Institute for Global Health, The University of Oxford, Hayes House, 75 George Street, Oxford, UK; 3 Department of Public Health, Walter Sisulu University, Eastern Cape, South Africa; 4 Center for Community Healthcare, Research and Development, Abuja, Nigeria; 5 Faculty of Health Sciences, Durban University of Technology, Durban, South Africa; 6 Department of Public Health, Faculty of Health Sciences, University of Fort Hare, Eastern Cape, South Africa

**Keywords:** Maternal health, Antenatal care, HIV test, Global health, Gambia

## Abstract

**Background:**

Improving the coverage of antenatal care is regarded as an important strategy to reduce the risks of maternal and child mortality in low income settings like Gambia. Nonetheless, a large number of countries in Africa, including Gambia, are struggling to attain an optimum level of healthcare utilization among pregnant women. The role of socioeconomic inequalities in maternal healthcare uptake has received little attention in Gambia. To address this evidence gap, the present study analyses nationally representative data to explore the socioeconomic inequalities in the use of maternal healthcare.

**Methods:**

Data on women aged 15–49 years (*n* = 5351) were extracted from the latest round of Gambia Demographic and Health Survey in 2013 for this study. The outcome measures were early and adequate antenatal visit and HIV tests during the last pregnancy. Data were analyzed using descriptive and multivariate regression methods. Socioeconomic status was assessed through the women’s education, type of employment, and household wealth quintile.

**Results:**

From the total of 5351 participants included in the study, 38.7 and 78.8% of the women had early and adequate ANC visits respectively with a 65.4% HIV test coverage during ANC visits. The odds of early [OR = 1.30, 95% confidence interval (CI) =1.06, 1.59] and adequate [OR = 1.45, 95%CI = 1.15, 1.82] ANC visits were higher in the rural areas compared with urban. Women with secondary [OR = 1.24, 95%CI = 1.04, 1.48] and higher education [OR = 1.80, 95%CI = 1.20, 2.70] had higher odds of making early ANC visits. Women from richest wealth quintile households had significantly higher odds of having early [OR = 1.49, 95%CI = 1.14, 1.95] and adequate ANC visits [OR = 2.06, 95%CI = 1.48, 2.87], but not of having HIV tests. Having access to electronic media showed a positive association with adequate ANC visits [OR = 1.32, 95%CI = 1.08, 1.62] and with taking HIV test during ANC [OR = 1.48, 95%CI = 1.21, 1.80]. A fewer odds of having unintended child was associated with early ANC visit [OR = 0.70, 95%CI = 0.59, 0.84], but positively associated with taking HIV test [OR = 1.75, 95%CI = 1.42, 2.15].

**Conclusion:**

A large proportion of women in Gambia were not using antenatal care and HIV tests during pregnancy. There are important sociodemographic differences in using maternal healthcare services such as HIV testing during pregnancy. This calls for strategic direction to promote the utilization of these services.

## Background

Globally, the low-middle-income countries altogether account for nearly all pregnancy and childbirth related deaths (99%) with sub-Saharan Africa alone making up 66% of the global total [1]. The burden is unacceptably high given the increasing availability of low-cost prevention measures provisioned through the basic maternal health care services that are provided by skilled childbirth attendant, and postnatal services. To reduce the maternal and child associated mortality, the World Health Organization (WHO) provided recommendations called to guide routine clinic visits by pregnant women. These clinics visits to nurture the wellbeing of mother and child during pregnancy by skilled health professionals is termed ‘antenatal care (ANC)’ [2].

Antenatal care (ANC) offers a distinctive platform that ensures a healthy pregnancy through; promotion of healthy weight gain and proper nutrition (e.g. intake of iron and folic acid), encouraging the uptake of necessary immunization packages such as tetanus toxoid and antimalarial drugs, controlling gestational diabetes, hypertension, and other pregnancy-related medical conditions [3–5]. Through ANC visits, pregnant women can easily follow up on the growth of the fetus, their own health including HIV status with focus on prevent mother-to-child transmission of HIV [6–8], a key contributor to childhood HIV in Africa [9, 10].

Women seeking ANC are also predicted to utilize postnatal care satisfactorily. Postnatal visits represent another important opportunity for prevention of mother to child transmission (PMTCT) of HIV during breastfeeding which is the safest infant feeding option among mothers in low-income settings. Antenatal care (ANC) is also an important predictor of using health facility delivery services that offer hygienic conditions and life-saving equipment which are vital for controlling the risks of delivery complications [1, 11]. The postnatal period represents another critical phase in the lives of mothers and newborns as most maternal and infant deaths take place during this period. Nonetheless, a large proportion of the women in most African countries like in Gambia are unable to access basic antenatal and postnatal care services, leading to child delivery outside healthcare settings under unsafe and unhygienic conditions [10].

Some studies have investigated various factors associated with maternal healthcare-seeking behavior such as behavioral, cultural, economic, and sociodemographic at different levels including individual, access to health facilities, inadequate infrastructure and skilled human resources for community healthcare [12–16]. Gambia does not, however, have such a study or report available, hence factors influencing low utilization of ANC services are unknown. To address this gap, our study examined factors associated with the use of ANC services using nationally representative data from Gambia Demographic and Health Survey (DHS) conducted in 2013 [17]. The Gambia DHS was conducted using a cross-sectional study design and it has information on a variety of demographic and socioeconomic variables.

## Methods

### Data source

Data analyzed to achieve the objective of this was obtained from the Gambia Demographic and health survey (GDHS) conducted in 2013 [17, 18]. The survey was implemented by Gambia Bureau of Statistics (GBOS) and the Ministry of Health and Social Welfare [17, 18]. The main purpose of DHS surveys is to provide quality information for monitoring and evaluation of population health programmes and assist in evidence-based health policy making. For this survey, sample population were selected from 14 sampling stratum divided into 281 Enumeration Areas (EAs) or clusters (also known as primary sampling units) throughout the eight regions (known as Local Government Areas). DHS surveys used multistage sampling strategy for sample selection. In the first stage, the EAs were selected with probability proportional to size and with independent selection in each sampling stratum. After selection of the EAs, 25 households in each EA were selected using equal probability systematic selection. A total of 105 interviewers and supervisors were recruited for training and the training was conducted from November 26 to December 14 of 2012. Data collection for the survey took place from February 2 to April 28 of 2013. A total of 10,233 women were interviewed with a response rate of 90.7%. Further details of the surveys are available from the final report by GBOS [17].

### Outcome measures

The study had three outcomes variables, and these were: 1) timing of first antenatal care, 2) frequency of antenatal care, 3) HIV testing during ANC visit. Determination of these outcome variables was based on the participant’s self-report for the latest childbirth that occurred within the last 5 years of the survey. The ANC visits were categorized as ‘timely’ if within the first trimester and ‘late’ if beyond the first trimester [1]. The frequency of ANC visits was defined as adequate (at least four visits) and inadequate (less than four visits) as recommended by the World Health Organization recommendation at the time the data used for this study was collected during the survey [19]. During the ANC visits, HIV testing was categorized as Yes (had HIV tests done) and No (had no HIV tests done).

### Independent variables

The independent variables identified and included in the analysis was based upon the availability of key variables and plausible covariates in the dataset. These variables include:

Predisposing factors: Age (15-19 years, 20-24 years, 25-29 years, 30-34 years, 35-39 years, 40-44 years, and 45-49 years); Residency (Urban, Rural); Religion (Islam, Other); Ethnicity Mandinka/Jahanka, Wollof Jola/Karoninka, Fula/Tukulur/Lorobo, Serahuleh Other); Parity (1–5, > 5); Household head (Male, Female); Child wanted (Wanted Then, Wanted No More); Enabling factors: Education (No Education, Primary, Secondary, Higher); Husband’s education (No Education, Incomplete Primary, Incomplete Secondary, Higher); Employment (Not Working, Professional/Technical/Managerial, Agricultural - Self Employed); Wealth quintile (Poorest, Poorer, Middle, Richer, Richest); Access to electronic media (No, Yes); Need factor: Heard of FP on internet (No, Yes);

### Data analysis

We used Stata version 14 to analyze the data. Data was cleaned, coded and analyzed based on the study inclusion criteria of at least 1 childbirth experience in the past 5 years. Since the survey that provided this data used cluster sampling techniques, all analyses were adjusted to the effect with the *svy* command [20]. This command uses data on the sampling weight, strata, and primary sampling units that are given with the dataset. The characteristics of the study population were described as percentages. Prevalence of timing and adequacy of antenatal care were presented as bar charts. This was followed by the use of binary logistic regression models to estimate the odds ratio (with 95%CIs) of using these services. Separate tables were used to present results of the three outcome variables, each divided into three subsamples: overall, urban and rural. Following the regression analysis, the model fitness was evaluated using the variance inflation factor (VIF) command. No multi-collinearity was detected as VIF values were below 10 for all the models. The preformed statistical test was two-tailed with the significant alpha value set at 5%.

## Results

### Sample description

As shown in Table 1, a larger proportion of the participants were; aged 25–29 years (25.40%), rural residents (63.61%), had no literate schooling (61.69%), had no jobs (41.39%), had access to electronic media (87.85%) from households with poorer wealth quintile (24.76%), didn’t hear about family planning on internet (98.88%), followers of Islam (98.02%), of Mandinka/Jahanka ethnicity (31.87%), had 1–5 children (76.28%), from male-headed households (82.88%), and wanted the last child (84.75%).

**Table 1:** Sample characteristics

Age	N = 5351	%
15–19	370	6.91
20–24	1176	21.98
25–29	1359	25.40
30–34	1129	21.10
35–39	786	14.69
40–44	391	7.31
45–49	140	2.62
**Residency**		
Urban	1947	36.39
Rural	3,44	63.61
**Education**		
No Education	3,31	61.69
Primary	762	14.24
Secondary	1144	21.38
Higher	144	2.69
**Husband’s education**		
No Education	3459	67.30
Incomplete Primary	284	5.53
Incomplete Secondary	1087	21.15
Higher	310	6.03
**Occupation**		
Not Working	2205	41.39
Professional/Technical/Managerial	1276	23.95
Agricultural - Self Employed	1847	34.67
**Wealth index**		
Poorest	1264	23.62
Poorer	1325	24.76
Middle	1128	21.80
Richer	850	15.88
Richest	784	14.65
**Media access**		
No	645	12.15
Yes	4665	87.85
**Heard about FP on internet**		
No	5283	98.88
Yes	60	1.12
**Religion**		
Islam	5239	98.02
Other	106	1.98
**Ethnicity**		
Mandinka/Jahanka	1688	31.87
Wollof	747	14.10
Jola/Karoninka	397	7.49
Fula/Tukulur/Lorobo	1,39	26.24
Serahuleh	416	7.85
Other	659	12.44
**Parity**		
1 to 5	4082	76.28
> 5	1269	23.72
**Sex of household head**		
Male	4435	82.88
Female	916	17.12
**Wanted last child**		
Wanted Then	4525	84.75
Wanted No More	814	15.25

**Fig. 1:**
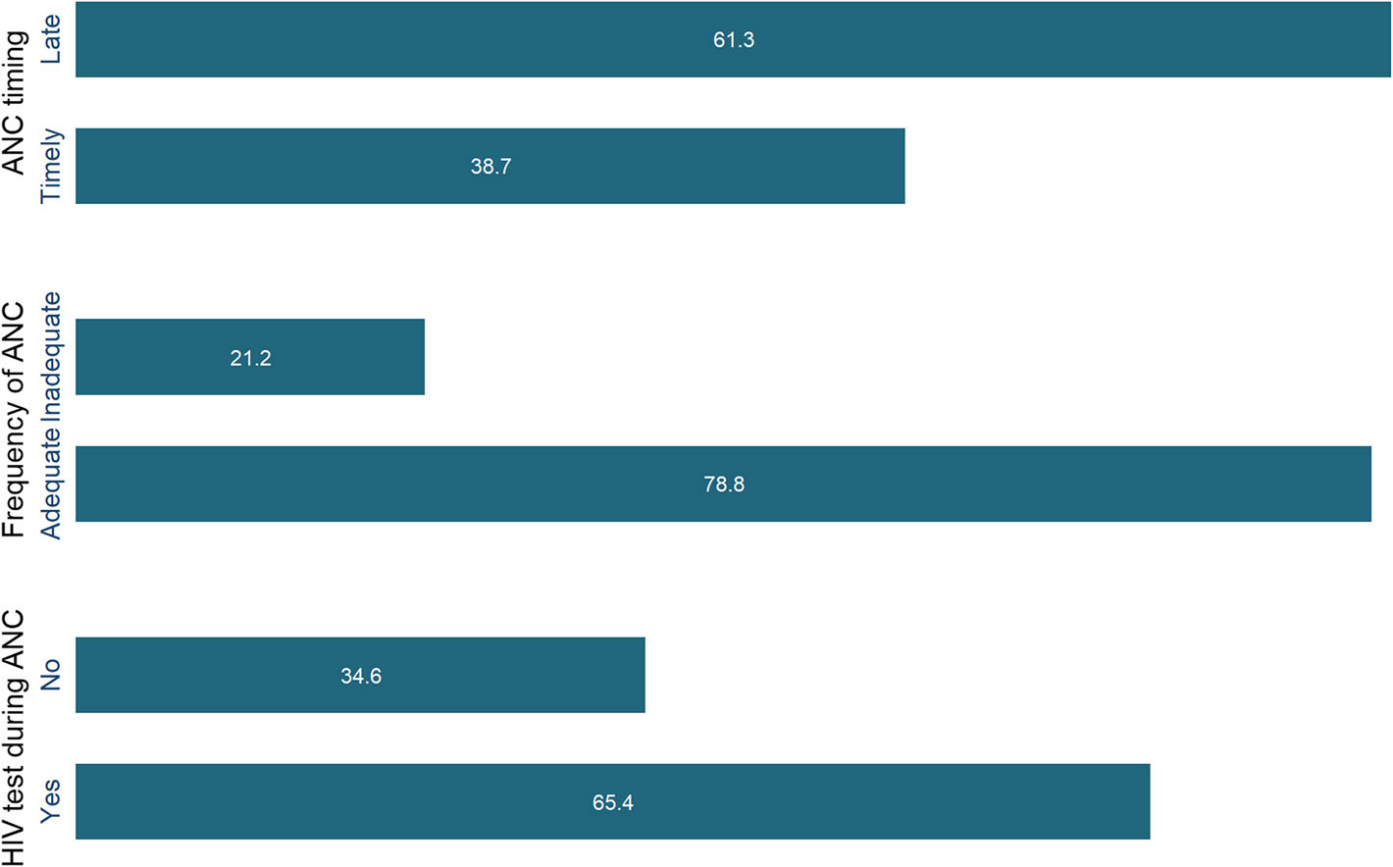
Percentage of women receiving early and adequate ANC and HIV test

Figure 1 shows that less than two-fifth made early contact, nearly two-thirds had an HIV test during ANC and a majority had adequate number of ANC visits. Precisely, the prevalence of women who had early, and adequate ANC visits were respectively 38.70% (*N* = 2071) and 78.80% (*N* = 4217). Nearly two-third (65.40%, *N* = 3500) of the women received HIV test during ANC visits.

The sociodemographic predictors of early and adequate ANC visit were presented in Tables 2 and 3 respectively. Compared with women in the lowest age group, those aged 20–24 years had lower odds of having early [‘Odds Ratio (OR)’=0.63, 95% ‘confidence interval (CI)’= 0.47,0.86] and adequate [OR = 0.68, 95%CI = 0.47, 0.98] ANC visits in the rural areas. No significant difference was observed in the urban areas. The odds of early [OR = 1.30, 95%CI = 1.06,1.59] and adequate [OR = 1.45, 95%CI = 1.15, 1.82] ANC visits were higher in the rural areas compared with urban. However, women in rural areas had lower odds of receiving HIV tests during ANC visits (Table 4). Women with secondary [OR = 1.24, 95%CI = 1.04, 1.48] and higher education [OR = 1.80, 95%CI = 1.20, 2.70] had higher odds of making early ANC visits. The educational difference was not significant for adequate ANC visits, but for HIV test it showed a strong positive association [OR = 1.45, 95%CI = 1.15,1.84]. Having employment showed a 45% increased association with early and adequate ANC visits, but a 19% decreased association with HIV test [OR = 0.81, 95%CI = 0.67,0.97]. Women from richest wealth quintile households had significantly higher odds of having early [OR = 1.49, 95%CI = 1.14,1.95] and adequate ANC visits [OR = 2.06, 95%CI = 1.48,2.87], but not of having HIV tests. Having access to electronic media showed a positive association with adequate ANC visits [OR = 1.32, 95%CI = 1.08, 1.62] and with taking HIV test during ANC [OR = 1.48, 95%CI = 1.21,1.80]. women from Wollof ethnicity had lower odds of having early and adequate ANC tests, but higher odds of having HIV tests [OR = 1.50, 95%CI = 1.20, 1.87]. Higher parity (> 5 children) showed 17% lesser odds of association with early [OR = 0.83, 95%CI = 0.69,0.99] and adequate [OR = 0.71, 95%CI = 0.57,0.89] ANC visits. Having unintended child had lesser odds of association [OR = 0.70, 95%CI = 0.59, 0.84] compared with early ANC visit, but increased odds with taking HIV test [OR = 1.75, 95%CI = 1.42, 2.15].

**Table 2:** Predictors of making early ANC visits in Gambia (n = 5351)

	Overall	Urban	Rural
**Age (15–19)**	1	1	1
20–24	0.71^*^ [0.55,0.92]	0.96 [0.57,1.63]	0.63^**^ [0.47,0.86]
25–29	0.97 [0.75,1.26]	1.11 [0.66,1.86]	0.96 [0.71,1.29]
30–34	1.00 [0.77,1.31]	1.35 [0.80,2.29]	0.87 [0.63,1.20]
35–39	1.07 [0.80,1.44]	1.37 [0.79,2.40]	0.96 [0.67,1.37]
40–44	1.09 [0.77,1.53]	1.64 [0.85,3.16]	0.89 [0.59,1.34]
45–49	1.04 [0.66,1.63]	1.06 [0.43,2.65]	0.99 [0.58,1.69]
**Residency (Urban)**	1	NA	NA
Rural	1.30^*^ [1.10,1.59]		
**Education (No Education)**	1	1	1
Primary	1.18 [0.99,1.40]	0.93 [0.68,1.26]	1.33^**^ [1.08,1.65]
Secondary	1.24^*^ [1.04,1.48]	1.28 [1.00,1.65]	1.15 [0.89,1.48]
Higher	1.80^**^ [1.20,2.70]	1.58 [1.00,2.51]	2.37 [0.88,6.38]
**Husband’s education (No Education)**	1	1	1
Primary	1.21 [0.94,1.57]	1.29 [0.87,1.92]	1.19 [0.85,1.68]
Secondary	1.07 [0.91,1.26]	1.11 [0.87,1.42]	1.05 [0.83,1.32]
Higher	1.18 [0.90,1.55]	1.42 [0.99,2.03]	0.93 [0.61,1.43]
**Employment (Not Working)**	1	1	1
Professional/Technical/Managerial	1.03 [0.88,1.21]	1.11 [0.90,1.38]	0.92 [0.71,1.18]
Agricultural - Self Employed	1.33^***^ [1.14,1.54]	1.52^*^ [1.02,2.25]	1.24^*^ [1.04,1.47]
**Wealth quintile (Poorest)**	1	1	1
Poorer	1.05 [0.89,1.25]	0.72 [0.38,1.35]	1.10 [0.92,1.31]
Middle	1.02 [0.85,1.22]	0.92 [0.55,1.53]	1.03 [0.85,1.26]
Richer	1.05 [0.83,1.33]	0.89 [0.56,1.42]	0.93 [0.65,1.33]
Richest	1.49^**^ [1.14,1.95]	1.14 [0.71,1.84]	4.78^*^ [1.25,1.83]
**Access to electronic media (No)**	1	1	1
Yes	0.92 [0.77,1.11]	1.17 [0.70,1.95]	0.88 [0.72,1.08]
**Heard of FP on internet (No)**	1	1	1
Yes	1.85^*^ [1.05,3.26]	1.57 [0.72,3.43]	2.18 [0.95,5.00]
**Religion (Islam)**	1	1	1
Other	1.15 [0.71,1.87]	1.48 [0.77,2.86]	0.74 [0.35,1.58]
**Ethnicity (Mandinka/Jahanka)**	1	1	1
Wollof	0.66^***^ [0.54,0.80]	0.85 [0.61,1.19]	0.56^***^ [0.43,0.72]
Jola/Karoninka	1.17 [0.92,1.49]	1.11 [0.79,1.58]	1.24 [0.88,1.77]
Fula/Tukulur/Lorobo	1.11 [0.95,1.30]	1.06 [0.79,1.42]	1.09 [0.90,1.31]
Serahuleh	1.10 [0.87,1.39]	1.24 [0.77,2.00]	1.00 [0.76,1.32]
Other	1.17 [0.95,1.44]	1.21 [0.91,1.60]	1.19 [0.86,1.63]
**Parity (1–5)**	1	1	1
> 5	0.83^*^ [0.69,0.99]	0.73 [0.53,1.02]	0.91 [0.73,1.13]
**Household head (Male)**	1	1	1
Female	0.99 [0.84,1.17]	1.10 [0.88,1.36]	0.88 [0.68,1.14]
**Child wanted (Wanted Then)**	1	1	1
Wanted No More	0.70^***^ [0.59,0.84]	0.85 [0.64,1.12]	0.61^***^ [0.48,0.77]
Pseudo *R*^2^	0.02	0.03	0.03

Exponentiated coefficients; 95% confidence intervals in brackets

^*^p < 0.05, ^**^p < 0.01, ^***^p < 0.001

**Table 3:** Predictors of making adequate ANC visits in Gambia

	Overall	Urban	Rural
**Age (15–19)**	1	1	1
20–24	0.74 [0.54,1.00]	0.91 [0.51,1.63]	0.68^*^ [0.47,0.98]
25–29	0.93 [0.68,1.27]	1.13 [0.64,2.01]	0.85 [0.59,1.24]
30–34	1.23 [0.89,1.71]	1.50 [0.83,2.71]	1.12 [0.75,1.67]
35–39	1.34 [0.93,1.94]	1.76 [0.92,3.36]	1.16 [0.74,1.83]
40–44	1.64^*^ [1.06,2.53]	2.20 [0.99,4.88]	1.44 [0.85,2.44]
45–49	1.54 [0.88,2.72]	1.02 [0.39,2.69]	1.91 [0.93,3.92]
**Residency (Urban)**	1	NA	NA
Rural	1.45^**^ [1.15,1.82]		
**Education (No Education)**	1	1	1
Primary	1.20 [0.96,1.49]	1.21 [0.84,1.75]	1.16 [0.88,1.52]
Secondary	1.07 [0.86,1.32]	1.17 [0.86,1.60]	0.94 [0.70,1.28]
Higher	0.89 [0.52,1.54]	1.04 [0.55,1.96]	0.46 [0.14,1.56]
**Husband’s education (No Education)**	1	1	1
Primary	1.01 [0.74,1.38]	0.90 [0.57,1.43]	1.10 [0.71,1.69]
Secondary	1.04 [0.85,1.28]	0.95 [0.71,1.27]	1.09 [0.82,1.45]
Higher	1.42 [0.98,2.05]	1.23 [0.76,2.00]	1.72 [0.96,3.11]
**Employment (Not Working)**	1	1	1
Professional/Technical/Managerial	1.26^*^ [1.03,1.53]	1.22 [0.94,1.58]	1.30 [0.95,1.76]
Agricultural - Self Employed	1.22^*^ [1.02,1.45]	0.98 [0.61,1.56]	1.22 [1.00,1.49]
**Wealth quintile (Poorest)**	1	1	1
Poorer	1.16 [0.95,1.41]	0.76 [0.38,1.51]	1.21 [0.99,1.49]
Middle	1.09 [0.89,1.35]	0.70 [0.40,1.23]	1.17 [0.93,1.48]
Richer	1.36^*^ [1.04,1.79]	0.95 [0.56,1.63]	1.32 [0.85,2.04]
Richest	2.06^***^ [1.48,2.87]	1.40 [0.80,2.44]	1.31 [0.28,6.22]
**Access to electronic media (No)**	1	1	1
Yes	1.32^**^ [1.08,1.62]	1.48 [0.88,2.51]	1.28^*^ [1.02,1.61]
**Heard of FP on internet (No)**	1	1	1
Yes	2.22 [0.87,5.67]	1.54 [0.45,5.26]	3.14 [0.72,13.65]
**Religion (Islam)**	1	1	1
Other	2.20 [0.93,5.19]	1.37 [0.51,3.65]	6.75 [0.90,50.73]
**Ethnicity (Mandinka/Jahanka)**	1	1	1
Wollof	0.62^***^ [0.50,0.76]	0.89 [0.60,1.32]	0.52^***^ [0.40,0.68]
Jola/Karoninka	0.95 [0.70,1.29]	0.91 [0.60,1.40]	1.01 [0.64,1.60]
Fula/Tukulur/Lorobo	0.95 [0.79,1.15]	0.87 [0.62,1.23]	0.97 [0.77,1.22]
Serahuleh	1.12 [0.82,1.51]	0.94 [0.52,1.72]	1.15 [0.80,1.64]
Other	0.98 [0.76,1.27]	1.02 [0.72,1.45]	0.92 [0.62,1.37]
**Parity (1–5)**	1	1	1
> 5	0.71^**^ [0.57,0.89]	0.61^*^ [0.41,0.91]	0.78 [0.59,1.02]
**Household head (Male)**	1	1	1
Female	1.16 [0.94,1.43]	1.19 [0.90,1.57]	1.12 [0.82,1.55]
**Child wanted (Wanted Then)**	1	1	1
Wanted No More	1.01 [0.82,1.23]	0.93 [0.66,1.30]	1.06 [0.81,1.37]
Pseudo *R*^2^	0.03	0.03	0.03

Exponentiated coefficients; 95% confidence intervals in brackets

^*^p < 0.05, ^**^p < 0.01, ^***^p < 0.001

**Table 4:** Predictors of HIV testing during ANC visits in Gambia

	Overall	Urban	Rural
**Age (15–19)**	1	1	1
20–24	1.21 [0.91,1.59]	1.23 [0.69,2.21]	1.17 [0.85,1.61]
25–29	1.33^*^ [1.01,1.75]	1.31 [0.74,2.34]	1.30 [0.94,1.78]
30–34	1.34^*^ [1.00,1.79]	1.38 [0.76,2.51]	1.26 [0.90,1.77]
35–39	1.23 [0.88,1.71]	1.57 [0.82,3.01]	1.04 [0.70,1.54]
40–44	1.22 [0.82,1.82]	1.42 [0.65,3.13]	1.12 [0.70,1.79]
45–49	1.18 [0.68,2.04]	2.13 [0.64,7.12]	0.89 [0.47,1.68]
**Residency (Urban)**	1		
Rural	0.64^***^ [0.51,0.81]	NA	NA
**Education (No Education)**	1	1	1
Primary	1.30^*^ [1.06,1.58]	0.99 [0.69,1.43]	1.45^**^ [1.15,1.84]
Secondary	1.05 [0.86,1.28]	1.02 [0.74,1.39]	1.05 [0.80,1.37]
Higher	1.03 [0.61,1.76]	0.87 [0.47,1.58]	5.82 [0.73,46.65]
**Husband’s education (No Education)**	1	1	1
Primary	0.94 [0.70,1.25]	0.81 [0.51,1.28]	1.03 [0.71,1.50]
Secondary	1.13 [0.94,1.37]	1.21 [0.89,1.63]	1.06 [0.83,1.36]
Higher	1.37 [0.99,1.91]	1.07 [0.68,1.69]	1.65^*^ [1.00,2.70]
**Employment (Not Working)**	1	1	1
Professional/Technical/Managerial	0.81^*^ [0.67,0.97]	0.73^*^ [0.56,0.95]	0.90 [0.68,1.18]
Agricultural - Self Employed	0.87 [0.74,1.03]	0.51^**^ [0.32,0.81]	1.02 [0.85,1.22]
**Wealth quintile (Poorest)**	1	1	1
Poorer	1.07 [0.89,1.27]	0.98 [0.49,1.94]	1.08 [0.90,1.31]
Middle	1.16 [0.95,1.41]	1.21 [0.68,2.18]	1.16 [0.94,1.43]
Richer	1.17 [0.90,1.52]	1.24 [0.72,2.13]	1.17 [0.80,1.72]
Richest	1.07 [0.78,1.46]	1.06 [0.61,1.85]	2.20 [0.57,8.53]
**Access to electronic media (No)**	1	1	1
Yes	1.48^***^ [1.21,1.80]	1.72 [0.98,3.02]	1.45^***^ [1.17,1.80]
**Heard of FP on internet (No)**	1	1	1
Yes	1.26 [0.61,2.62]	0.60 [0.23,1.55]	3.00 [0.86,10.49]
**Religion (Islam)**	1	1	1
Other	1.56 [0.81,2.99]	2.85^*^ [1.04,7.84]	1.09 [0.45,2.65]
**Ethnicity (Mandinka/Jahanka)**	1	1	1
Wollof	1.50^***^ [1.21,1.87]	1.07 [0.71,1.62]	1.77^***^ [1.36,2.31]
Jola/Karoninka	1.78^***^ [1.30,2.45]	1.33 [0.83,2.13]	2.17^***^ [1.39,3.38]
Fula/Tukulur/Lorobo	0.96 [0.81,1.13]	0.88 [0.62,1.26]	1.02 [0.84,1.25]
Serahuleh	1.15 [0.88,1.50]	1.35 [0.72,2.53]	1.14 [0.85,1.54]
Other	1.00 [0.78,1.25]	0.69^*^ [0.49,0.98]	1.40 [0.98,2.00]
**Parity (1–5)**	1	1	1
> 5	1.02 [0.83,1.26]	0.72 [0.49,1.08]	1.16 [0.91,1.48]
**Household head (Male)**	1	1	1
Female	0.91 [0.75,1.10]	0.84 [0.64,1.11]	1.02 [0.77,1.36]
**Child wanted (Wanted Then)**	1	1	1
Wanted No More	1.75^***^ [1.42,2.15]	0.99 [0.71,1.40]	2.29^***^ [1.77,2.98]
Pseudo *R*^2^	0.03	0.03	0.03

Exponentiated coefficients; 95% confidence intervals in brackets

^*^p < 0.05, ^**^p < 0.01, ^***^p < 0.001

## Discussion

Optimal utilization of maternal healthcare services is essential for ensuring better pregnancy outcome and consequently the health of future generations. A healthy pregnancy determines not only the health of the newborn, but also has a major impact on adult health with broader and long-lasting implications for national human development efforts. Data driven evidence is therefore necessary to inform current initiatives on maternal and child health (MCH) to improved maternal health outcomes as set out in the sustainable development goals (SDGs). Our study proffer information on the prevalence and predictors of the three key components of maternal healthcare including early and adequate ANC visits and utilization of HIV testing services among pregnant women in Gambia. The results show that in the first trimester, the proportion of women who made their first ANC visit was 38.70% whereas about four-fifth (78.80%) of the women had adequate ANC visits. In a multi-country study that examined data from the demographic and health surveys, the prevalence of making adequate ANC visits was as follows; Cameroon (62.70%), Senegal (51.20%), and Uganda (48.50%) [20]. Notably was that data on the timing of the ANC visits were less regularly documented. A nationally-representative survey in Ethiopia showed that 33.7% of the sample population made early ANC visits [1] which correlates with results from the present study. Regarding HIV tests during pregnancy, a more recent Ethiopian study found that 35.10% of the sample population were tested for HIV compared with 65.4% in the present study [21]. However, the prevalence of Gambian women taking HIV tests during pregnancy is lower compared with Uganda (81.50%) and Mozambique (69.40%) [22].

In the multivariable analysis, significant sociodemographic differences were observed in the likelihood of the utilization of the ANC services. Although women’s age is generally a strong predictor of using healthcare services [7], in our study the age difference was found to be significant for those in the age group of 20–24 years. Women in this age group had lower odds of using timely and adequate ANC services. It is however important to note that the difference was significant in the rural areas only. Strangely, the odds of early and adequate ANC visits were higher in the rural areas compared with urban. The general consensus is that the prevalence of healthcare use is more prevalent among urban population compared with their rural counterparts due to better health awareness, financial capacity, transportation facilities. In a Nigerian study, for instance, women in the rural areas had lower likelihood of receiving maternal healthcare services compared with urban women. The causes behind the urban-rural disparity in the use of ANC service in Gambia needs further exploration.

Women with relatively higher education and wealth status were more likely to make early ANC visits and take HIV tests as anticipated. Thus, the educational status of women is a critical indicator of their social equality and a significant financial indicator of the ability to access healthcare services. Education can also influence women’s healthcare seeking behaviour through promoting health awareness and self-efficacy [23]. Women who are able to gather information regarding the health risks associated with pregnancy are presumably more enabled to have improved health seeking behaviors through proactive health risk reduction practices and management strategies that involves consulting qualified healthcare professionals such as specialized medical practitioners for diagnosis and treatment when sick. In addition, the advent of information and communication technology (ICT) has allowed ICT gadgets like TV, radio and ICT services like the internet to serve as source of knowledge that informs women’s use of healthcare, especially in the developing countries [24].

Nonetheless, education alone may not ensure adequate access to healthcare use as it’s a complex construct and is influenced by a multitude of economic and sociocultural factors. For instance, women in financially better-off families may enjoy better access to healthcare compared with educated ones with inadequate financial capacity. Moreover, cultural factors may shape people’s concept of health and illness and the perceived need for seeking professional care they may not be familiar with. Based on these insights, healthcare policy making is regularly suggested to address the financial and cultural barriers to accessing maternal healthcare in low-income settings. There is an indication of ethnic influence in accessing ANC services, but only limited to a few ethnic groups in this study, which might be indicative of a lesser influence of cultural factors in using healthcare services.

Having employment showed an increased likelihood of having early and adequate ANC visits, but lower likelihood of having an HIV test. Having an employment translates to better economic situation and can act an enabling factor for utilization of healthcare services. Maternal healthcare promotion programs should therefore pay special attention to non-working women who might remain deprived of the essential services owing to poor financial means. Non-working women are also more likely to have higher fertility and consequently share an increased risk of pregnancy related health complications including unintended pregnancies. In our study, having unintended child was found to lower the likelihood of having early ANC visit, but significantly increase the likelihood of taking a HIV test. This is presumably because of the lack of awareness and anxiety associated with the unplanned pregnancy that prevent women from taking active steps in ensuring an optimum gestational health. In the context of Gambia, the practice of routine HIV tests during pregnancy should be regarded as a special priority to keep the prevalence of Childhood HIV under control. Although the country has a relatively lower prevalence of HIV, maternal transmission of HIV remains a major risk factor and become a serious public health hazard unless necessary prevention and interventions steps are taken. Altogether, these findings strongly advocate for continued effort to improve women’s education, economic status, better fertility control, and health communication through mass media to facilitate the use of antenatal healthcare services and HIV testing in the Gambia.

This study is the first to report the prevalence of using ANC services in nationally-representative sample in Gambia. Important sociodemographic disparities were observed in the odds of using the services, among which the most notable were women’s education, wealth status of the household, parity, and family planning communication through mass media. These findings of the present study fill an important gap in the literature. Main strengths were the use of nationally representative data from four rounds of survey that allows making a generalizable conclusion about the prevalence and associations. Practical implications of the study include the role of the information in designing health policies. The findings provide potentially important information for promoting PMTCT programs in Gambia.

Nonetheless, this study has several limitations to report. First of all, the data was collected using a cross-sectional study design, hence no causality can be inferred from the associations [25]. Also, the study used a secondary data thus the authors had no influence over the selection and measurement of the variables. Lack of relevant data prevented us from exploring key questions such as late initiation of ANC care. As the data were self-reported, the chances of recall and reporting bias cannot be ignored. In 2013, when the data was collected, recommended cut-offs for adequate number of ANC visits was four (4) compared to present time (2020) where the recommendations has been updated by the world health organization to be eight (8) visits. The authors analyzed the 2013 Gambia national demographic health survey, though we are aware that the second and next national survey is scheduled for October 2019 to February 2020.

## Conclusion

Based on a nationally-representative sample, the present study showed that the proportion of women making their first ANC visit within the first trimester was about two-fifth, and nearly two-third of the women received HIV test during ANC visits. About four-fifth of the women met the World Health Organization’s recommendation of taking at least four ANC visits. Important sociodemographic disparities were observed in the odds of using the services, among which the most notable were women’s education, wealth status of the household, parity, and exposure to family planning communication. With regards to ethnic background, there was a slight indication of ethnicity influence on uptake of ANC services but this was only limited to a few ethnic groups (particularly Wollof compared to Jahanka). Barriers to ANC services utilization and HIV testing during pregnancy highlighted by the socioeconomic and cultural inequalities should be given special attention in designing intervention programs.

## Data Availability

Data for this study were sourced from Gambia Demographic and Health surveys (DHS) and available here: https://dhsprogram.com/data/available-datasets.cfm

## Abbreviations

ANC: Antenatal care
DHS: Demographic Health Survey
EAs: Enumeration Areas
GBOS: Gambia Bureau of Statistics
HIV: Human Immunodeficiency Virus
LMIC: Low and middle-income countries
MCH: Maternal and child health
PMTCT: Prevention of Mother-to-Child Transmission
VIF: Variance inflation Factor
